# Dietary Inulin Supplementation Modulates Short-Chain Fatty Acid Levels and Cecum Microbiota Composition and Function in Chickens Infected With *Salmonella*

**DOI:** 10.3389/fmicb.2020.584380

**Published:** 2020-12-09

**Authors:** Jiao Song, Qinghe Li, Nadia Everaert, Ranran Liu, Maiqing Zheng, Guiping Zhao, Jie Wen

**Affiliations:** ^1^State Key Laboratory of Animal Nutrition, Institute of Animal Sciences, Chinese Academy of Agricultural Sciences, Beijing, China; ^2^Precision Livestock and Nutrition Unit, Gembloux Agro-Bio Tech, University of Liège, Gembloux, Belgium

**Keywords:** cecum microbiota, chicken, inulin, metagenome sequencing, *Salmonella*

## Abstract

The current study investigated the effects of inulin on the gut microbiota, microbiome functions, and short-chain fatty acids (SCFAs) levels in specific pathogen-free (SPF) chickens infected with *Salmonella* enteritidis (SE). SPF Arbor Acres chickens (*n* = 240, 1-day-old) were divided into four groups: a control group (CON) fed a basal diet without inulin supplementation or SE infection, and three groups fed a basal diet supplemented with inulin 0, 0.5, and 1% (SE, 0.5%InSE, 1%InSE, respectively) up to 28-days-old, followed by SE challenge at 28 days of age. Cecal SCFA contents and microbiome composition and function were analyzed at 1-day post-infection. The results showed that SE infection significantly decreased cecal butyrate concentrations compared with the CON group (*p* < 0.05), while inulin supplementation reversed these changes compared with the SE group (*p* < 0.05). Inulin supplementation at 1% significantly increased the abundances of *Lactobacillus* and *Streptococcus*, and significantly decreased the abundances of *Subdoligranulum* and *Sellimonas* compared with the SE group (*p* < 0.05). The functional profiles of microbial communities based on metagenomic sequencing analysis showed that SE infection significantly increased the abundances of pathways related to carbohydrate metabolism, amino acid metabolism, energy metabolism, metabolism of cofactors and vitamins, and glycan biosynthesis and metabolism (*p* < 0.05), and significantly decreased the abundances of pathways related to nucleotide metabolism, translation, and replication and repair compared with the CON group (*p* < 0.05), and these effects were reversed by inulin supplementation (0.5 and 1%) (*p* < 0.05). In conclusion, inulin modulated the dysbiosis induced by SE infection via affecting SCFA metabolism and microbial functional profiles.

## Introduction

Chicken meat is one of the most important and economical sources of animal protein for human consumption worldwide. However, the modern poultry industry uses large amounts of antimicrobials to prevent disease and enhance growth and productivity, resulting in antimicrobial resistance and a risk to human health (Shang et al., 2018). Numerous studies have demonstrated that many bacterial pathogens isolated from poultry meat, such as *Escherichia coli* and *Salmonella*, possess resistance genes (Abraham et al., 2019). *Salmonella* Enteritis (SE) infection in poultry leads to reduced growth and lower economic benefits, and poses a threat to food safety and human public health as a result of possible foodborne outbreaks in humans (Nair et al., 2018). Antibiotic-based growth promoters will consequently be totally banned in China in 2020. Alternatives to antibiotic growth promoters in animal feed are therefore receiving increasing attention, with the aim of improving meat quality and safety, while promoting animal productivity and welfare (Markowiak and Śliżewska, 2018).

Inulin-type fructans are one of the best-studied prebiotics and are used to improve poultry growth performance and meat quality (Guaragni et al., 2019), enhance nutrient utilization and immune function (Xia et al., 2019), regulate gut microbiota-related metabolism disorders, and alleviate intestinal inflammation (Singh et al., 2019). On one hand, inulin can be recognized directly by carbohydrate receptors on the surface of the intestinal epithelial cell and immune cells (such as dendritic cells), which consequently modulate the host immune response and inhibit pathogen colonization (Wu et al., 2017; Teng and Kim, 2018). Inulin has been reported to downregulate the gene expression of proinflammatory cytokines, such as tumor necrosis factor-α (TNF-α), interferon-γ, and interleukin (IL)-6, and inhibit pro-inflammatory immunity (Teng and Kim, 2018), which provides metabolic advantages to gut commensal bacteria to compete with *Salmonella* (Sun et al., 2018). On the other hand, inulin has indirect regulatory effects that modulate dysbiosis and the host immune response based on microbiota-dependent mechanisms (Sivaprakasam et al., 2016). Generally, inulin stimulates the growth and activity of lactic acid bacteria and can also be fermented by gut commensal bacteria, serving as a nutrient source for the bacteria, which generate short-chain fatty acids (SCFAs), mainly acetate, propionate, and butyrate. These in turn suppress infection by acid-sensitive pathogens through creating an acidic environment (Teng and Kim, 2018). The regulatory mechanisms of SCFAs have been studied extensively, including their functions in nutrition, energy metabolism, microbiota regulation, histone deacetylase inhibition, lysine deacetylase inhibition, G-protein coupled receptor activation, and acylation of bacterial virulence regulators (Zhang et al., 2019). However, most studies in poultry have focused on microbial metabolic regulation, especially the proliferation of beneficial microbiota such as *Bifidobacterium* and *Lactobacillus*, and the inhibition of colonization by pathogenic bacteria, such as *Salmonella* (Micciche et al., 2018).

The addition of inulin may thus provide a strategy for reducing the abundance of SE via a microbiota-dependent immunomodulatory mechanism. Indeed, SE infection potentially changes gut microbial communities in poultry, resulting in gut dysbiosis. SE challenge decreased the abundance of SCFA-producing *Clostridia, Bifidobacterium*, and *Lactobacillus*, leading to decreased SCFA levels, attenuating the intestinal innate immune response, and increasing gut inflammation (Rivera-Chávez et al., 2016). Inulin also increased the concentration of SCFAs, which in turn enhanced the abundance of the major beneficial bacteria *Bifidobacteria* and *Lactobacillus* in the cecum, which competed with pathogenic bacteria and reduced the SE populations (Micciche et al., 2018). The composition of the intestinal microbiota may play a fundamental role in the defensive mechanism of inulin against SE infection. However, the cecum is the most densely populated microbial habitat in the chicken gut (up to 10^11^ cells/g), but its microbial composition remains largely unknown (Stanley et al., 2013).

Therefore, the current study aimed to use metagenomics approaches to determine how SE infection affected SCFA production and functional microbial profiles in the chicken cecum, and to clarify how inulin modulated the dysbiosis induced by SE infection via SCFA metabolism and microbial functional profiles. These results will provide new opportunities for improving the gut microecosystem balance and reducing the presence of pathogens to maintain gut health and food safety.

## Materials and Methods

The study protocol was approved by the Animal Welfare Committee of the Institute of Animal Sciences (Chinese Academy of Agricultural Sciences, Beijing, China), Ethical approval regarding animal survival was given by the animal ethics committee of IAS-CAAS (approval number: IASCAAS-AE20140615).

### Animal Feeding and Management

A total of 240, 1-day-old Arbor Acres specific pathogen-free (SPF) chicks were acquired from a local hatchery (Merial Vital Laboratory Animal Technology Co., Ltd., Beijing, China). All birds were housed in a sterile isolation chamber (IPQ-Type 3 negative pressure isolator; China Agricultural University, Beijing, China) and fed a corn/soybean-based sterile diet on the day of arrival. The birds were randomly assigned to four groups (six replicates of 10 birds each): a control group (CON) fed a basal diet, and three groups fed with basal diet supplemented with different doses of inulin (0, 0.5, and 1%) up to 28 days of age, followed by challenge at day 28 with SE (SE, 0.5%InSE, and 1%InSE groups, respectively). The inulin with DP of >10 was obtained from *Cichorium intybus* (Sigma-Aldrich, St. Louis, MO, United States). Details of the basal diet ingredients and calculated nutrient contents are provided in Supplementary Table 1. The temperature in the isolator was maintained at 35°C for the first week and then decreased by 2°C each week until the end of the experiment (day 29). All chickens had free access to feed and water (sterilized at 121°C for 15 min).

### SE Challenge

*Salmonella* enteritidis (CMCC50041, China Institute of Veterinary Drugs Control, Beijing) was obtained from the China Center of Industrial Culture Collection. Briefly, chickens in the SE, 0.5%InSE, and 1%InSE groups were infected with 1.0 mL of an actively growing culture of SE of 1 × 10^8^ colony-forming units/mL by oral gavage at 28 days of age. Birds in the CON group received an equal volume of phosphate-buffered saline.

### Cecal Sample Collection

At 1-day post-infection, one chicken from each replicate was randomly selected and sacrificed. The cecum on both sides was opened using sterile scissors and tweezers, and the cecal contents were squeezed out into two frozen tubes and stored at −80°C for SCFA and DNA extraction for 16S rDNA and metagenomics analysis.

### SCFA Concentration Analysis

Cecal SCFA concentrations were measured by gas chromatography-mass spectrometry. Briefly, cecal contents (50 mg) were homogenized in 200 μL of 24% metaphosphoric acid using a vortex mixer for 15 min and centrifuged at 5,000 × *g* for 5 min at 4°C. The supernatants were analyzed using a DB-FFAP column (Agilent Technologies, Santa Clara, CA, United States) and mass-selective flame ionization detector (FID) with the following parameters: injector volume, 2 μL; split ratio, 50:1; injector temperature, 250°C; FID temperature, 280°C; and carrier gas, helium. The initial oven temperature was 70°C for 10 min, increased by 5°C/min to 210°C, and then maintained for 12 min. The acetic acid, propionic acid, and butyric acid peaks in a standard solution (Sigma-Aldrich, MO, United States) were analyzed using the same parameters. The peaks of individual SCFAs in each cecal sample were acquired using the same parameters. The molar concentration of each SCFA was calculated using the ratio of the peak area of the individual SCFA and the peak area of the standard solution multiplied by the concentration of the standard solution.

### High-Throughput Sequencing of the Microbial 16S rRNA Gene

The cecal microbiota was determined with 16S rRNA sequencing analysis as described previously (Tan et al., 2019). Briefly, bacterial DNA was extracted from 22 cecal digested samples (5 from CON and SE group respective and 6 from 0.5%InSE, and 1%InSE groups respective) using an E.Z.N.A.^®^ Stool DNA Kit (Omega Bio-tek, Norcross, GA, United States) according to the manufacturer’s protocols. The DNA concentration and purification were determined using TBS-380 and a NanoDrop 2000 UV-vis spectrophotometer (Thermo Scientific, Wilmington, DE, United States), respectively. DNA quality was examined by 1% agarose gel electrophoresis. The V3-V4 hypervariable regions of the bacterial 16S rRNA gene were amplified with primers 338F (5′-ACTCCTACGGGAGGCAGCAG-3′) and 806R (5′-GGACTACHVGGGTWTCTAAT-3′) using a thermocycler polymerase chain reaction (PCR) system (GeneAmp 9700, ABI, CA, United States). The resulting PCR products were extracted from a 2% agarose gel and further purified using an AxyPrep DNA Gel Extraction Kit (Axygen Biosciences, Union City, CA, United States) and quantified using QuantiFluor^TM^-ST (Promega, WI, United States) according to the manufacturer’s protocol. Purified amplicons were pooled in equimolar amounts and subjected to paired-end sequencing on an Illumina MiSeq platform (Illumina, San Diego, CA, United States) according to standard protocols, by Majorbio Bio-Pharm Technology Co., Ltd. (Shanghai, China).

### Processing of Sequencing Data and Diversity Analysis

Raw fastq files were demultiplexed and quality-filtered to obtain high quality clean tags according to QIIME (V1.7.0; San Diego, CA, United States) with the following criteria (Magoč and Salzberg, 2011): (i) 300 bp reads were truncated at any site with an average quality score <20 over a 50 bp sliding window, discarding truncated reads <50 bp; (ii) exact barcode matching, a two nucleotide mismatch in primer matching and reads containing ambiguous characters were removed; and (iii) only sequences with >10 bp overlap were assembled according to their overlap sequence. Reads that could not be assembled were discarded. Operational taxonomic units (OTUs) were clustered with 97% similarity cut-off using UPARSE (version 7.1^1^), and chimeric sequences were identified and removed using the UCHIME algorithm. The taxonomy of each 16S rRNA gene sequence was analyzed by RDP Classifier^2^ against the Silva (SSU115) 16S rRNA database, with a confidence threshold of 70%. Alpha and beta diversity were analyzed based on these output normalized data. Chao1, Simpson, Shannon, and ACE indexes were calculated to assess the alpha-diversity in each sample using QIIME (V1.7.0; San Diego, CA, United States) and displayed using R software (Version 3.3.1, R Core Team, Vienna, Austria). Beta diversity based on unweighted UniFrac was performed to differentiate among the samples in terms of species complexity using QIIME software (V1.7.0; San Diego, CA, United States). Principal coordinates analysis (PCoA) was applied to reduce the dimension of the original variables and executed with QIIME and R software using the unweighted pair-group method with arithmetic means.

### Library Construction and Metagenomics Sequencing

The cecal microbiota was further investigated using a metagenomic sequencing method as described previously (Yang et al., 2017). DNA was extracted from 12 samples (3 from each group respective) for metagenomics analysis, using an E.Z.N.A.^®^ Stool DNA Kit (Omega Bio-tek, Inc., Norcross, GA, United States) according to the manufacturer’s protocols. DNA from each sample was then fragmented to an average size of approximately 300 bp using Covaris M220 (Gene Company Limited, Beijing, China) for paired-end library construction, using a TruSeq^TM^ DNA Sample Prep Kit (Illumina Inc., San Diego, CA, United States). Adapters containing the full complement of sequencing primer hybridization sites were ligated to the blunt-end fragments. Paired-end sequencing was performed on an Illumina HiSeq4000 platform (Illumina Inc., San Diego, CA, United States) at Majorbio Bio-Pharm Technology Co., Ltd., using a HiSeq 3000/4000 PE Cluster Kit and HiSeq 3000/4000 SBS Kits, according to the manufacturer’s instructions^3^.

### Sequence Quality Control and Genome Assembly

Reads were aligned to the *Gallus gallus* genome (version 5.0) by BWA^4^ and hits associated with the reads and their paired reads were removed. The 3′ and 5′ ends were stripped using SeqPrep^5^. Low quality reads (length < 50 bp, quality value < 20, or having N bases) were removed using Sickle^6^. De Bruijn-graph-based assembler SOAP *de novo* (^7^ Version 1.06) was employed to assemble short reads (Li et al., 2008). K-mers, varying from 1/32/3 of read length, were tested for each sample. Scaffolds > 500 bp were retained for statistical tests. The quality and quantity of scaffolds generated by each assembly were evaluated and the K-mer that yielded the minimum scaffold number and the maximum values of N50 and N90 was finally selected. Scaffolds > 500 bp were extracted and broken into contigs without gaps and the contigs were used for further gene prediction and annotation.

### Gene Prediction, Taxonomy, and Functional Annotation

Open reading frames (ORFs) from each metagenomic sample were predicted using Metagene^8^. Predicted ORFs ≥ 100 bp were retrieved and translated into amino acid sequences using the NCBI translation table^9^. All sequences from gene sets with a 95% sequence identity (90% coverage) were clustered as the non-redundant gene catalog using CD-HIT^10^. After quality control, reads were mapped to the representative genes with 95% identity using SOAP aligner (see text footnote 7), and the gene abundance in each sample was evaluated. Taxonomic annotations were carried out using BLASTP (Version 2.2.28+^11^) by aligning non-redundant gene catalogs against the NCBI NR database, with an e-value cut-off of 1e^–5^. Kyoto Encyclopedia of Genes and Genomes (KEGG) pathway analysis was conducted using BLASTP search (Version 2.2.28+) against the KEGG database^12^, with an e-value cut-off of 1e^–5^. Based on the comparison results, functional annotation was performed using KOBAS 2.0 (KEGG language-based annotation system). The corresponding gene abundance sum of KEGG orthologies (KOs) and KEGG were then used to calculate the abundance of the corresponding functional category.

### Statistical Analysis

Data are shown as mean ± standard deviation. Differences in SCFA contents, alpha diversity indices, relative abundance of bacterial taxa among treatment groups, and bacterial functional pathways were analyzed using SAS 9.2 (SAS Institute, Cary, NC, United States), followed by Duncan’s multiple comparison tests. Differences between two groups were analyzed using Student’s *t*-test. A significant difference was declared when *p* < 0.05. Spearman’s correlations between SCFA contents and the top 15 significantly different genera of cecal microbiota were assessed using the PROC CORR procedure in SAS 9.2. A correlation matrix was created and visualized in a heatmap format using R package version 3.3.1. Cecal microbiota abundance and SCFA concentration were considered to be correlated with each other when the absolute value of the correlation coefficient (r) was > 0.55 and *p* < 0.05. PCoA, hierarchical clustering analysis, and heat map analyses were conducted using R package version 3.3.1.

## Results

### Inulin Affects the Production of SCFA

Cecal digesta SCFA levels are shown in Table 1. SE challenge significantly decreased the butyrate content and increased the acetate and propionate contents (*p* < 0.05) of the cecal digesta compared with the CON group, while inulin supplementation (0.5 and 1%) significantly increased acetate and butyrate concentrations (*p* < 0.05), and 1% inulin supplementation decreased the propionate concentration compared with the SE group (*p* < 0.05).

**TABLE 1:** Effects of inulin on cecal SCFA contents in SE-infected chickens (mmol/kg).

Item	Treatment^1^				*p-*value
	
	CON	SE	0.5% InSE	1% InSE	
Acetate	4.09 ± 0.58^d^	5.29 ± 0.40^c^	8.04 ± 0.56^b^	9.36 ± 0.52^a^	<0.001
Propionate	0.26 ± 0.02^c^	0.50 ± 0.05^a^	0.48 ± 0.04^a^	0.38 ± 0.02^b^	<0.001
Butyrate	3.34 ± 0.15^c^	2.88 ± 0.44^d^	4.37 ± 0.37^b^	6.38 ± 0.58^a^	<0.001

*^*a*–*d*^Means within rows with different superscript letters were significantly different (*p* < 0.05).*

*^1^CON: basal diet without inulin supplementation; SE: *Salmonella*-infected chicken fed with basal diet; 0.5% InSE: *Salmonella*-infected chicken fed with 0.5% inulin; 1% InSE: *Salmonella*-infected chicken fed with 1% inulin.*

### Analysis of the Microbial 16S rRNA High-Throughput Sequencing

The 16S rRNA gene sequencing was performed to analyze the cecal microbial community structures in the different groups. After assigning OTUs and removing chimeras, there were 35,000–50,000 sequencing reads in each sample (mean 44,845 ± 3,390), with a mean read length of 433 bp (range 427–439) (Supplementary Table 2). A total of 372 OTUs, eight phyla, and >90 genera and 160 species were identified based on >97% sequencing similarity (Supplementary Table 3), with 266 OTUs shared by all four groups (Supplementary Figure 1). Good’s coverage index (Supplementary Table 2) showed that sufficient sequencing coverage was acquired.

Alpha diversity richness indexes (Sobs, Chao1, ACE) and diversity indexes (Shannon and Simpson) of the cecal microbiota are shown in Table 2. The Shannon index was significantly higher in the CON and inulin supplementation groups than in the SE group (*p* < 0.05), and the Sobs, ACE, and Chao1 indexes were significantly higher in inulin supplementation groups than in the CON group (*p* < 0.05).

**TABLE 2:** Alpha diversity of cecal microbiota.

Item	CON^1^	SE	0.5%InSE	1%InSE	*p-*value
Sobs	21818.61^b^	24028.64^ab^	26221.30^a^	25516.03^a^	0.0161
ACE	238.2517.57^b^	262.7526.60^ab^	285.9918.89^a^	270.6717.79^a^	0.0093
Chao1	23421.94^c^	26327.03*b*^c^	29723.43^a^	27219.41^ab^	0.0028
Shannon	3.420.18^a^	3.070.16^b^	3.430.15^a^	3.440.24^a^	0.0115
Simpson	0.080.02	0.120.03	0.090.02	0.090.05	0.2981

*^*a*–*c*^Means within rows with different superscript letters were significantly different (*p* < 0.05).*

*^1^CON: basal diet without inulin supplementation; SE: *Salmonella*-infected chicken fed with basal diet; 0.5% InSE: *Salmonella*-infected chicken fed with 0.5% inulin; 1% InSE: *Salmonella*-infected chicken fed with 1% inulin.*

**FIGURE 1:**
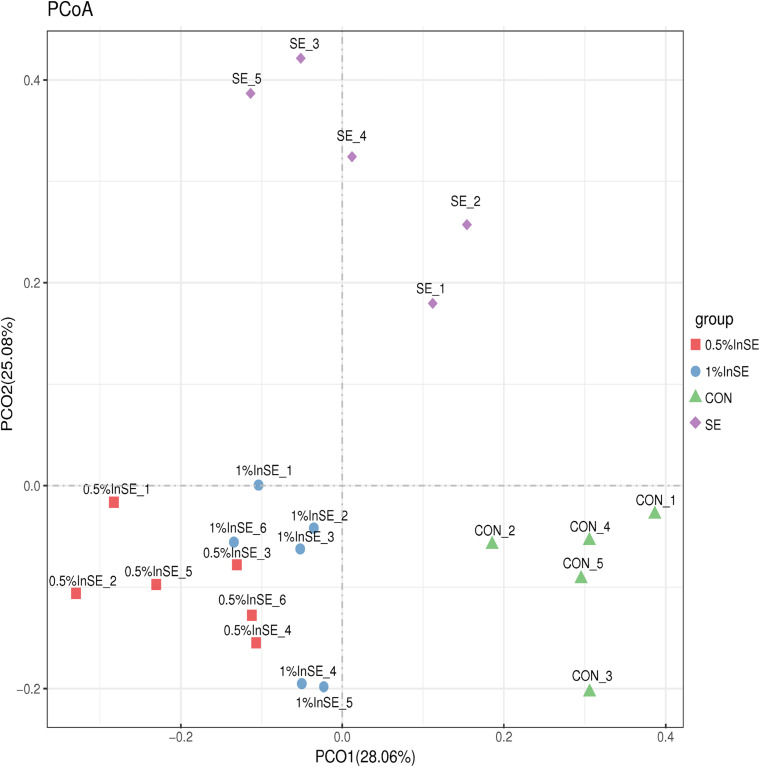
Comparison of the compositions of the cecal microbiota by principal coordinate analysis (PCoA).

The beta diversity analysis was measured by unweighted UniFrac distances and illustrated in a PCoA plot, which was used to estimate the similarity among different groups (Figure 1). Similar to PCoA, clustering analysis revealed short UniFrac distances between the two inulin groups and long distances between the samples and the CON, and SE and inulin-treated groups (Supplementary Figure 2). The dissimilarity in cecal microbial communities was greatest between the SE and inulin groups.

**FIGURE 2:**
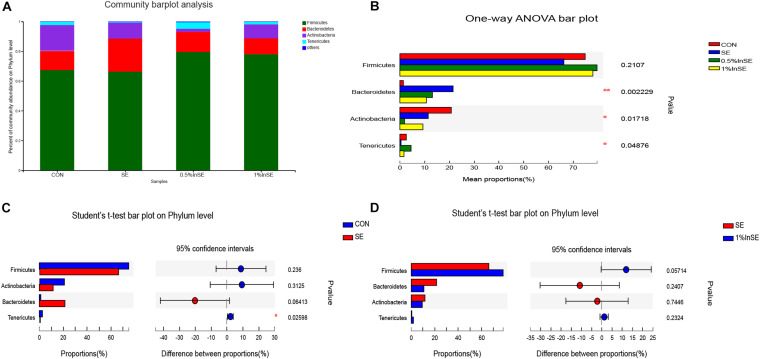
Relative abundance of cecal microbiota phyla by 16s profiling. CON (*n* = 5): basal diet without inulin supplementation; SE (*n* = 5): *Salmonella*-infected chicken fed with basal diet; 0.5% InSE (*n* = 6): *Salmonella*-infected chicken fed with 0.5% inulin; 1% InSE (*n* = 6): *Salmonella*-infected chicken fed with 1% inulin. **(A)** Relative abundance of cecal microbiota phyla for CON, SE, 0.5%InSE and 1%InSE group. **(B)** The cecal microbiota showed differential abundance at the phylum level among four groups according to one-way ANOVA. **(C)** The cecal microbiota showed differential abundance at the phylum level between the CON and SE groups according to *t*-tests. **(D)** The cecal microbiota showed differential abundance at the phylum level between the SE and 1%InSE groups according to *t*-test.

The most abundant phyla are presented in Figure 2A. The dominant phyla in the cecal microbiota across all the groups were Firmicutes (73.6–84.9%), Bacteroidetes (0.87–24.4%), Actinobacteria (0.49–22.6%), and Tenericutes (0.5–3.2%), which accounted for >99% of the total sequences (Figure 2A). SE infection alone resulted in a lower abundance of Firmicutes compared with the CON and inulin-supplementation groups, but the difference was not significant (*p* = 0.2107). However, SE challenge and inulin supplementation significantly altered the relative abundance of Bacteroidetes (*p* = 0.0022), Actinobacteria (*p* = 0.0171), and Tenericutes (*p* = 0.0487) (Figure 2B). SE challenge increased the abundance of Bacteroidetes (*p* = 0.06) and decreased the abundance of Actinobacteria (*p* = 0.3125) and Tenericutes (*p* = 0.0259) compared with the CON group (Figure 2C). However, the addition of 1% inulin increased the abundance of Firmicutes (*p* = 0.057) and decreased the abundance of Bacteroidetes (*p* = 0.241) compared with the SE group (Figure 2D).

**FIGURE 3:**
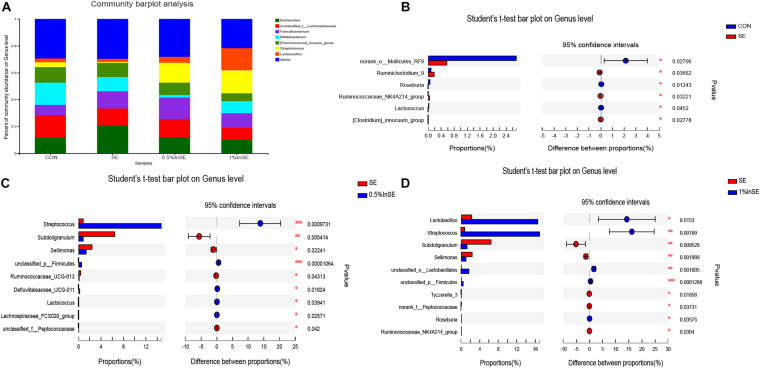
Relative abundance of cecal microbiota genera by 16s profiling. CON (*n* = 5): basal diet without inulin supplementation; SE (*n* = 5): *Salmonella*-infected chicken fed with basal diet; 0.5% InSE (*n* = 6): *Salmonella*-infected chicken fed with 0.5% inulin; 1% InSE (*n* = 6): *Salmonella*-infected chicken fed with 1% inulin. **(A)** Relative abundance of cecal microbiota genera for CON, SE, 0.5%InSE and 1%InSE groups. **(B)** The cecal microbiota showed differential abundance at the genus level between CON and SE group according to *t*-test. **(C)** The cecal microbiota showed differential abundance at the genus level between the SE and 0.5%InSE groups according to *t*-test. **(D)** The cecal microbiota showed differential abundance at the genus level between the SE and 1%InSE groups according to *t*-test.

A total of 95 bacterial genera were identified, of which *Lachnospiraceae*, *Faecalibacterium, Bifidobacterium*, *Bacteroides*, *Lactobacillus*, *Ruminococcus_torques_group*, and *Streptococcus* were the most abundant, accounting for 70.5–78.1% (Figure 3A). Among the top 15 genera in the cecal microbiota (Supplementary Figure 3), there were significant differences in the abundances of *Bacteroides*, *Bifidobacterium*, *Streptococcus, Subdoligranulum*, and *Sellimonas* among all groups. The differential abundances of the genera were analyzed by Student’s *t*-tests (Figures 3B–D). SE infection significantly reduced the abundances of *Mollicutes_RF* (*p* = 0.027), *Lactococcus* (*p* = 0.045), and *Roseburia* (*p* = 0.012), and increased the abundances of *Ruminiclostridium_9* (*p* = 0.037), *Clostridium_innocuum_group* (*p* = 0.028), and *Ruminococcaceae_*NK4A214*_group* (*p* = 0.032) among the top 15 genera, compared with the CON group (Figure 3B). Inulin supplementation (1 and 0.5%) significantly increased the abundances of *Streptococcus* and *Firmicutes* and significantly decreased the abundances of *Subdoligranulum* and *Sellimonas* compared with the SE-infection group (*p* < 0.05) (Figures 3C,D). Moreover, inulin supplementation at 0.5% significantly increased the abundances of *Defluviitaleaceae_UCG-011* (*p* = 0.0184), *Lactococcus* (*p* = 0.039), and *Lachnospiraceae_FCS020_group* (*p* = 0.026) and decreased the abundances of *Ruminococcaceae_UCG-013* (*p* = 0.043) and *Peptococcaceae* (*p* = 0.042) (Figure 3C). Compared with the SE group, inulin supplementation at 1% also significantly increased the abundances of *Lactobacillus* (*p* = 0.0153), *Lactobacillales* (*p* = 0.001), and *Roseburia* (*p* = 0.0358), and significantly decreased the abundances of *Ruminococcaceae_NK4A214_group* (*p* = 0.0304), *Peptococcaceae* (*p* = 0.0373), and *Tyzzerella_3* (*p* = 0.0186) (Figure 3D). Inulin supplementation at 1% also significantly increased the abundances of *Lactobacillus* (*p* = 0.0314) and *Bifidobacterium* (*p* = 0.0210) compared with 0.5% inulin (Supplementary Figure 4).

### Correlations Among Microbiota Species and SCFA Metabolism

**FIGURE 4:**
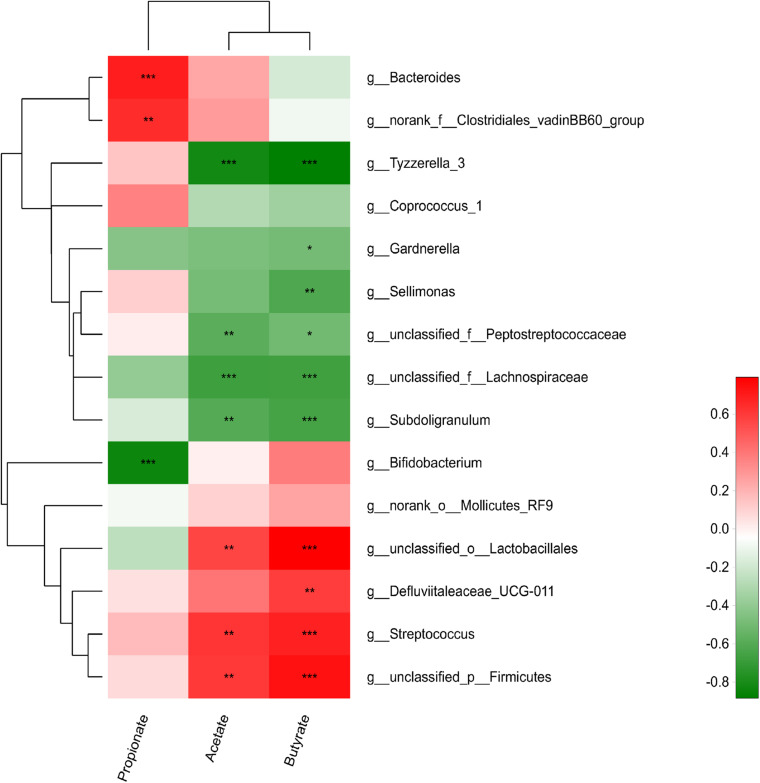
Correlations between relative abundances of cecal species and SCFA concentration. Red positive correlation; green, negative correlation; *strong correlation (| r| > 0.55, *p* < 0.05).

We further analyzed the association between the cecal microbiota and cecal SCFA by correlation analyses comparing the bacterial genera and cecal SCFA contents (Figure 4). *Streptococcus, Firmicutes*, and *Lactobacillales* were significantly positively correlated with acetate and butyrate contents (*r* > 0.55, *p* < 0.05), *vadinBB60, Defluviitaleaceae_UCG-011* and *Bacteroides* were significantly positively correlated with propionate and butyrate contents, respectively (*r* > 0.55, *p* < 0.05), while *Subdoligranulum*, *Lachnospiraceae, Tyzzerella_3*, and *Peptostreptococcaceae* were significantly negatively correlated with acetate and butyrate contents (*r* < −0.55, *p* < 0.05). *Bifidobacterium, Gardnerella*, and *Sellimonas* were significantly negatively correlated with propionate, acetate and butyrate contents, respectively (*r* < −0.55, *p* < 0.05).

### Functional Capacity Profiling of Cecum Microbiome Based on Metagenomic Sequencing

**FIGURE 5:**
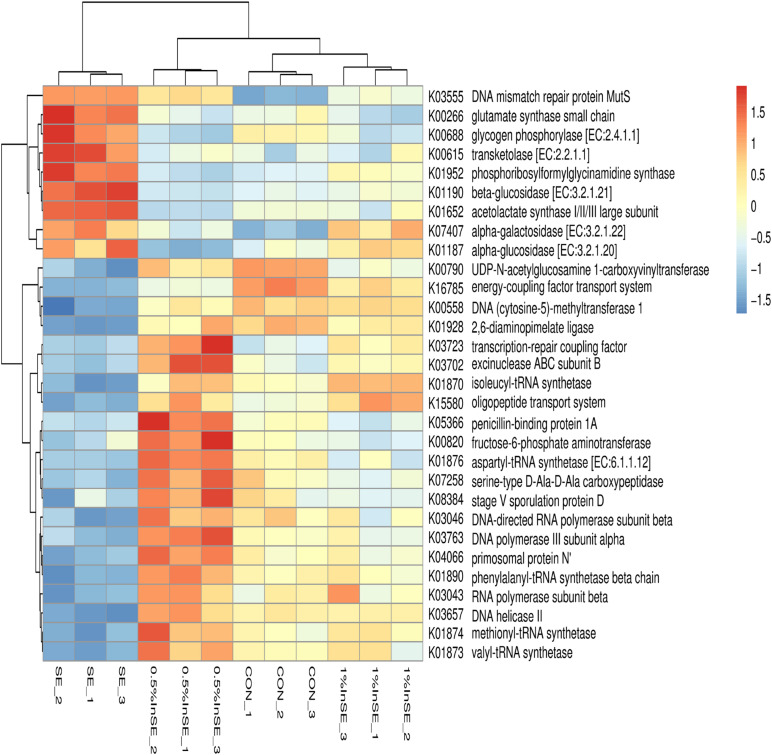
Hierarchical clustering analysis and heat map analysis of relative abundance of top 30 KEGG Orthologies (KOs) in the CON, SE, 0.5%InSE and 1%InSE groups. Red, high KO abundance; blue, low KO abundance. CON (*n* = 3): basal diet without inulin supplementation; SE (*n* = 3): *Salmonella*-infected chicken fed with basal diet; 0.5% InSE (*n* = 3): *Salmonella*-infected chicken fed with 0.5% inulin; 1% InSE (*n* = 3): *Salmonella*-infected chicken fed with 1% inulin.

The functional capacity of the gut microbiome was investigated using metagenomic sequencing data. Based on the annotation of ORFs predicted from the assembled contigs, a total of 8,608,302 ORFs with an average length of 595.64 bp was found to infer with the functional capacity. The predicted genes were classified by aligning them to the KEGG database. A total of 6725 KOs were identified and assigned to 402 KEGG pathways. A total of 1341 differential KOs between different treatments were analyzed, and the relative abundances of the top 30 KOs showed different enrichments (Figure 5) (*p* < 0.05). Compared with the CON group, nine of these 30 KOs were enriched in the SE group (*p* < 0.05), including KOs associated with carbohydrate metabolism (K00615, K01190, K07407, K01187, K00688), amino acid metabolism (K00266, K01652), nucleotide metabolism (K01952), and replication and repair (K03555). The changes in these nine KOs were reversed in the inulin-fed groups compared with the SE group (*p* < 0.05). The other 21 KOs had lower abundances in the SE group compared with the CON group (*p* < 0.05), such as carbohydrate metabolism (K00790, K00820), membrane transport (K16785, K15580), glycan biosynthesis and metabolism (K07258, K08384, K05366), replication and repair (K03657, K03723, K03702, K04066), amino acid metabolism (K00558, K01928), nucleotide metabolism (K03046, K03043, K03763), and translation (K01870, K01874, K01873, K01876, K01890) (Figure 5). These changes were also reversed by inulin supplementation compared with the SE group (*p* < 0.05).

**FIGURE 6:**
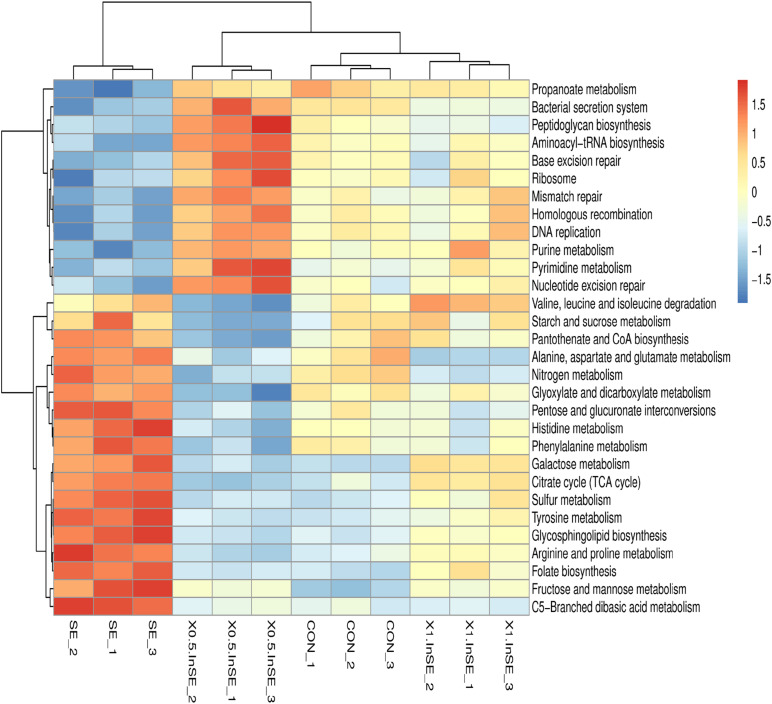
Hierarchical clustering analysis and heat map analysis on the relative abundance of KEGG microbial function at level-3 pathways in the CON, SE, 0.5%InSE and 1%InSE groups. Red, high KO abundance; blue, low KO abundance. CON (*n* = 3): basal diet without inulin supplementation; SE (*n* = 3): *Salmonella*-infected chicken fed with basal diet; 0.5% InSE (*n* = 3): *Salmonella*-infected chicken fed with 0.5% inulin; 1% InSE (*n* = 3): *Salmonella*-infected chicken fed with 1% inulin.

We further identified the top15 KEGG microbial functions at level 2 of the KEGG pathways terms showing distinct enrichments between inulin-supplemented chickens with and without SE infection (Table 3). The abundances of pathways related to carbohydrate metabolism, energy metabolism, metabolism of cofactors and vitamins, and glycan biosynthesis and metabolism were significantly increased in the SE group compared with the CON group (*p* < 0.05), while pathways related to nucleotide metabolism, translation, and replication and repair were significantly decreased (*p* < 0.05). However, the abundance of amino acid metabolism pathways was similar in the CON and SE groups. Inulin supplementation (0.5 and 1%) significantly reversed the changes in abundances of these KEGG pathways (*p* < 0.05). We also identified the top 30 differently enriched KEGG microbial functions and compared the KEGG pathways at level 3 (Figure 6 and Table 4). The abundances of 12 KEGG functional pathways, including one pathway in membrane transport (“Bacterial secretion system”), one in carbohydrate metabolism (“Propanoate metabolism”), one in glycan biosynthesis and metabolism (“Peptidoglycan biosynthesis”), five in replication and repair (“Homologous recombination,” “DNA replication,” and “Mismatch/Base excision/Nucleotide excision”), two in translation (“Aminoacyl-tRNA biosynthesis” and “Ribosome”), and two pathways in nucleotide metabolism (“Purine/Pyrimidine metabolism”) were significantly reduced in the SE group compared with the CON group, while inulin addition significantly increased the abundances of these KEGG pathways compared with the SE group (*p* < 0.05). However, a total of 18 KEGG pathways, including seven pathways in carbohydrate metabolism (“Starch and sucrose/Galactose/Fructose and mannose/C5-Branched dibasic acid metabolism,” “Citrate cycle (TCA cycle),” and “Pentose and glucuronate interconversions”), one in glycan biosynthesis and metabolism (“Glycosphingolipid biosynthesis”), six pathways in amino acid metabolism (“Alanine. aspartate and glutamate/Histidine/Arginine and proline/Phenylalanine/Tyrosine metabolism/Valine, leucine and isoleucine degradation”), two in energy metabolism (“Nitrogen/Sulfur metabolism”), and two in metabolism of cofactors and vitamins (“Pantothenate and CoA/Folate biosynthesis”) were over-represented in the SE group compared with the CON group (*p* < 0.05), but were significantly reduced in the 0.5%InSE and 1%InSE groups compared with the SE group (*p* < 0.05).

**TABLE 3:** Main microbial pathways grouped into level-2 KEGG functional categories using PICRUSt.

Item	CON^1^	SE	0.5%InSE	1%InSE	*p-*value
Carbohydrate metabolism	15.750.12^c^	16.730.12^a^	15.020.22^d^	16.090.12^b^	0.0001
Amino acid metabolism	9.820.49^a^	9.680.34^a^	8.910.13^b^	9.590.21^a^	0.037
Nucleotide metabolism	6.230.06^b^	5.960.13^c^	6.590.08^a^	6.350.13^b^	0.0006
Membrane transport	6.240.04	5.680.74	6.260.09	6.040.18	0.2892
Energy metabolism	6.000.08^ab^	6.230.19^a^	5.750.11^c^	5.920.03^bc^	0.0076
Translation	5.570.05^b^	4.970.01^c^	6.170.20^a^	5.520.28^b^	0.0003
Replication and repair	5.440.09^b^	4.900.14^c^	5.950.11^a^	5.500.24^b^	0.0003
Metabolism of cofactors and vitamin	4.890.03^b^	5.460.16^a^	4.810.09^b^	5.010.09^b^	0.0002
Cellular community prokaryotes	3.490.12	3.270.24	3.410.05	3.230.17	0.2587
Lipid metabolism	3.120.02^b^	3.310.13^a^	3.230.08^ab^	3.210.06^ab^	0.1186
Glycan biosynthesis and metabolism	2.350.06^b^	2.790.23^a^	2.530.04^b^	2.300.05^b^	0.0046
Signal transduction	2.340.13^b^	2.350.10^b^	2.700.02^a^	2.370.02^b^	0.0019
Folding sorting and degradation	1.980.05^b^	2.020.04^b^	2.230.05^a^	2.020.07^b^	0.0018
Biosynthesis of other secondary metabolites	2.150.15^ab^	2.250.05^a^	1.770.03^c^	2.050.08^b^	0.001
Metabolism of other amino acids	1.870.06^ab^	1.970.07^a^	1.790.04^b^	1.960.07^a^	0.0187

*^*a*–*d*^Means within rows with different superscript letters were significantly different (*p* < 0.05).*

*^1^CON: basal diet without inulin supplementation; SE: *Salmonella*-infected chicken fed with basal diet; 0.5% InSE: *Salmonella*-infected chicken fed with 0.5% inulin; 1% InSE: *Salmonella*-infected chicken fed with 1% inulin.*

**TABLE 4:** The main microbial pathways grouped into level-3 KEGG functional categories using PICRUSt.

KEGG pathways	CON^1^	SE	0.5%InSE	1%InSE	*p-*value
**MEMBRANE TRANSPORT**					
Bacterial secretion system	0.880.001^b^	0.810.01^d^	0.900.01^a^	0.850.001^c^	<0.0001
**CARBOHYDRATE METABOLISM**					
Starch and sucrose metabolism	2.140.09^a^	2.240.07^a^	1.930.01^b^	2.160.09^a^	0.0057
Galactose metabolism	1.310.01^c^	1.570.04^a^	1.310.02^c^	1.480.01^b^	<0.0001
Fructose and mannose metabolism	0.850.01^c^	1.120.04^a^	0.940.01^b^	0.940.02^b^	<0.0001
Glyoxylate and dicarboxylate metabolism	0.820.01^b^	0.850.01^a^	0.760.01^c^	0.800.01^b^	<0.0001
Propanoate metabolism	0.850.02^a^	0.730.01^b^	0.840.01^a^	0.830.008^a^	<0.0001
Citrate cycle (TCA cycle)	0.610.01^c^	0.730.009^a^	0.570.004^d^	0.680.004^b^	<0.0001
Pentose and glucuronate interconversions	0.670.03^b^	0.780.01^a^	0.600.02^c^	0.640.02^c^	<0.0001
C5-Branched dibasic acid metabolism	0.310.01^b^	0.450.01^a^	0.310.01^b^	0.300.01^b^	<0.0001
**GLYCAN BIOSYNTHESIS AND METABOLISM**					
Peptidoglycan biosynthesis	1.550.05^b^	1.350.03^d^	1.820.08^a^	1.440.03^c^	<0.0001
Glycosphingolipid biosynthesis	0.180.004^c^	0.250.01^a^	0.170.004^c^	0.200.003^b^	<0.0001
**AMINO ACID METABOLISM**					
Alanine. aspartate and glutamate metabolism	1.390.03^b^	1.430.005^a^	1.320.002^c^	1.310.004^c^	<0.0001
Histidine metabolism	0.610.006^b^	0.660.01^a^	0.570.01^b^	0.590.02^c^	<0.0001
Arginine and proline metabolism	0.470.004^b^	0.540.01^a^	0.460.004^c^	0.500.002^d^	<0.0001
Phenylalanine metabolism	0.250.01^b^	0.290.01^a^	0.220.01^b^	0.240.01^c^	0.0002
Tyrosine metabolism	0.190.01^c^	0.300.01^a^	0.190.01^c^	0.220.01^b^	<0.0001
**REPLICATION AND REPAIR**					
Homologous recombination	1.620.03^b^	1.430.05^c^	1.750.04^a^	1.630.08^b^	0.0006
Mismatch repair	1.320.04^b^	1.170.03^c^	1.460.02^a^	1.350.06^b^	0.0002
DNA replication	1.170.02^b^	1.010.03^c^	1.250.02^a^	1.170.06^b^	0.0005
Base excision repair	0.660.004^b^	0.610.01^c^	0.710.02^a^	0.650.03^b^	0.0005
Nucleotide excision repair	0.670.03^b^	0.610.03^c^	0.780.02^a^	0.690.01^b^	<0.0001
**ENERGY METABOLISM**					
Nitrogen metabolism	0.440.01^b^	0.460.01^a^	0.390.01^c^	0.400.003^c^	<0.0001
Sulfur metabolism	0.360.01^c^	0.450.01^a^	0.360.003^c^	0.400.02^b^	<0.0001
**TRANSLATION**					
Aminoacyl-tRNA biosynthesis	1.880.03^b^	1.650.06^c^	2.100.03^a^	1.850.06^b^	<0.0001
Ribosome	3.470.01^b^	3.080.16^c^	3.850.15^a^	3.470.20^b^	0.0028
**NUCLEOTIDE METABOLISM**					
Purine metabolism	3.380.02^b^	3.170.04^c^	3.540.01^a^	3.450.09^a^	0.0001
Pyrimidine metabolism	2.830.02^b^	2.730.03^c^	3.050.07^a^	2.890.05^b^	0.0001
**METABOLISM OF COFACTORS AND VITAMINS**					
Pantothenate and CoA biosynthesis	0.760.03^b^	0.800.01^a^	0.670.01^c^	0.750.02^b^	0.0005
Folate biosynthesis	0.380.01^c^	0.550.01^a^	0.390.004^c^	0.450.03^b^	<0.0001

*^*a*–*d*^Means within rows with different superscript letters were significantly different (*p* < 0.05).*

*^1^CON: basal diet without inulin supplementation; SE: Salmonella-infected chicken fed with basal diet; 0.5% InSE: Salmonella-infected chicken fed with 0.5% inulin; 1% InSE: Salmonella-infected chicken fed with 1% inulin.*

## Discussion

The cecum is an important region for microbial activity in the chicken gut, partly because the greatest density of microbial communities (up to 10^11^ cells/g) occurs here, the digesta remains in the cecum for a relatively long time (10–12h), and microorganisms reside here longer than in the small intestine (Hong et al., 2019). Moreover, the cecal microbiome plays important nutritional and immune roles, such as nitrogen recycling, providing vitamins, amino acids, and SCFA to their host, especially by supplying energy to the host and competitively inhibiting pathogenic infections (Sood et al., 2019). In the current study, we observed that the cecal microbiome was mainly composed of Firmicutes, Bacteroidetes, and Actinobacteria at the phylum level, in accordance with other studies (Ocejo et al., 2019). Although some researchers also showed that Proteobacteria and Tenericutes were common phyla in the chicken cecum, and the abundances of these phyla were low in the current study. Among the six most abundant genera, only *Bifidobacterium* belonged to the phylum Actinobacteria, while *Faecalibacterium, Bacteroides*, *Lactobacillus*, *Ruminococcus*, and *Streptococcus* all belonged to the Firmicutes phylum, showing that the predominant cecal bacteria were Firmicutes.

The chicken cecal microbiome has been found to be affected by various factors, including diet, sex, genotype, feed additives, housing conditions, and pathogen infection (Clavijo and Flórez, 2018). Notably, pathogenic infections, such as *Clostridium perfringens*, *Eimeria* species, and SE, resulted in significant changes in the cecal microbiota (Juricova et al., 2013). In the present study, the abundances of Firmicutes, Actinobacteria, and Tenericutes phyla were reduced by SE infection; however, the abundance of Firmicutes (mainly *Lactobacillus* and *Streptococcus*) was increased by inulin supplementation. *Bifidobacterium* and *Lactobacillus* are considered as beneficial bacteria in many animal species, because of their ability to produce natural bacteriocines. *Lactobacillus* has also been reported to produce organic acids (lactic and acetic acids) in poultry, to promote growth performance and combat infection by pathogens, such as *Salmonella*, *Shigella*, *Clostridium*, and *Listeria* (Yadav and Jha, 2019). *Bifidobacterium* also acts as an immunostimulant, as well as competing with pathogenic bacteria for cell adhesion sites and producing essential SCFAs for energy production by the host (Yan et al., 2017). Inulin-type fructan, which is an important dietary fiber, can modulate the gut microbial population, especially by increasing the abundances of *Bifidobacterium* and *Lactobacillus* and inhibiting pathogen proliferation (*E. coli* and *Salmonella*) by promoting a low pH in the cecum of broiler chickens (Mesa et al., 2017). We previously showed that dietary inulin supplementation protected the gut mucosa by improving immune responses, SCFA production, and intestinal morphology and suppressing the colonization of SE in SPF broiler chickens (Song et al., 2020). However, the effects of inulin on the intestinal microbiota in chickens requires further investigation.

In the present study, inulin increased the abundance of *Lactobacillus*, but 0.5% inulin decreased the abundance of *Bifidobacterium* compared with SE infection. Xia et al. (2019) also showed that inulin lowered the relative abundances of *Lactobacillus* and *Bifidobacterium* species at an early age, but subsequently increased their relative abundances. Moreover, as a commensal organism in chickens, the genus *Streptococcus* played a vital role in reducing the colonization and incidence of SE (Adhikari et al., 2019). In this study, inulin also increased the abundance of *Streptococcus*. Inulin may therefore competitively inhibit *Salmonella* colonization and strengthen the immune system partly because of its ability to increase the proliferation of beneficial microbiota, such as *Bifidobacteria, Lactobacilli*, and *Streptococcus*, in the gastrointestinal tract (Micciche et al., 2018).

Short-chain fatty acids, including acetate, propionate, and butyrate, are the end-products of fermentation of inulin by gut microbes and have been extensively investigated for their roles in ameliorating the intestinal mucosal barrier and suppressing intestinal inflammation via several different mechanisms (Yang et al., 2016). SE infection decreased the concentration of butyrate, possibly as a result of a reduction in the abundance of butyrate-producing *Clostridia* induced by SE (Rivera-Chávez et al., 2016). The present results also showed that acetate and butyrate were the main cecal SCFAs that contributed to the acidic environment in the gut, to suppress pathogen growth (Teng and Kim, 2018). Inulin supplementation increased acetate and butyrate levels in the present study, in accordance with our observations on the changes in SCFAs (Song et al., 2018). Butyrate was reported to be the main energy source for the proliferation of intestinal epithelium cells and to increase villus height (De Vadder et al., 2014). Moreover, an increase in butyrate may increase oxygen consumption by colonocytes, leading to inhibition of the proliferation and expansion of aerobic bacteria, such as SE (Byndloss and Baumler, 2018; Litvak et al., 2018). Numerous reports have shown that SCFAs, especially butyrate, stimulate the growth of *Bifidobacteria* and *Lactobacilli* in poultry, which in turn promote an increase in SCFAs, lowering the pH and enhancing the mucosal immune function and inhibiting the growth of pathogenic bacteria such as *Salmonella* and *E. coli* (Micciche et al., 2018).

In the present study, Clostridiales, including *Sellimonas, Peptococcaceae, Lachnospiraceae*, and *Subdoligranulum*, had a negative relationship with SCFA production, while *vadinBB60, Bacteroides*, and *Defluviitaleaceae_UCG-011* had positive relationships with SCFA production. Moreover, *vadinBB60* was positively related to propionate production, and some reports showed that it was also associated with butyrate production (Van den Abbeele et al., 2010). In accordance with previous studies, the current study found that inulin increased the abundances of *Streptococcus* and *Lactobacillus*, which were shown to be positively related to butyrate production (Yan et al., 2017). *Streptococcus*, Lactobacillales, and Firmicutes exhibited a positive relationship with acetate production in the present study, playing an important role in reducing the colonization and incidence of SE through reducing the pH and competitive exclusion (Adhikari et al., 2019).

The functional profiles of microbial communities based on metagenomic sequencing analysis showed that the most abundant functions were carbohydrate metabolism, amino acid metabolism, nucleotide metabolism, membrane transport, and energy metabolism with significant differences in level 2 KEGG pathways among the four treatments. We also showed that the abundances of functional pathways related to membrane transport (bacterial secretion system) and replication and repair (mismatch repair, DNA replication) were reduced, while amino acid metabolism, carbohydrate metabolism, and energy metabolism were increased in the SE group compared with the CON group, and there were significant differences in the level 3 KEGG pathways between the SE and CON groups. Membrane transport pathways, such as bacterial secretion, are essential for protein secretion, enhancing cell attachment, and for the survival of bacteria in the gut ecosystem (Green and Mecsas, 2016). Pilmis et al. (2020) demonstrated that the bacterial secretion system would be related to defending endogenous bacteria (Bacteroidetes and Proteobacteria) against the overgrowth of certain exogenous pathogenic bacteria species, such as SE. In the current study, the abundance of the bacterial secretion system pathway, including the associated KOs (K16785, K15580), was significantly reduced in the SE group compared with the CON group. Similarly, Mon et al. (2020) showed that the bacterial secretion system was down-regulated in SE-infected chickens, pointing toward a possible downshift in the ability of intestinal microbes for adhesion and attachment of mucosal cells. However, inulin supplementation significantly increased the abundance of the bacterial secretion system and the related KOs. These results suggested that inulin might play an important role in affecting the gut microbiota composition in birds challenged with SE through the microbial functional metabolism.

The present results also showed that amino acid metabolism and energy metabolism at level 3, including the related KOs (K00266, K01652), were enriched in the SE-infection group, indicating that pathways associated with nutrients and energy metabolism were activated to meet the nutrient and energy requirements of the cecal microbiome in SE-infected chickens. However, the gut microbiota shifts toward the utilization of less favorable residual peptides and proteins to gain energy (proteolytic fermentation) when SCFAs are insufficient to meet their energy needs for gut microbial growth and proliferation because of SE infection (Makki et al., 2018; Canfora et al., 2019). SE infection resulted in a decrease in butyrate production in the present study, which contributed to enrichment of amino-acid-metabolism-related pathways involving “Phenylalanine metabolism,” “Tyrosine metabolism,” and “Valine, leucine and isoleucine degradation” in the SE group. Notably, microbial proteolytic fermentation can produce branched-chain amino acids (e.g., valine, leucine, and isoleucine) and aromatic amino acids (e.g., tyrosine, phenylalanine, and tryptophan), which can be further metabolized by microbiota to meet the energy requirement (Canfora et al., 2019). We therefore speculated that microbial fermentation of amino acids may also provide sources for energy metabolism (nitrogen and sulfur metabolism), which could explain why energy metabolism-related pathways involving “nitrogen metabolism” and “sulfur metabolism” were enriched in the SE-infection group. Studies have therefore suggested that infected chickens should be supplied with the required energy by providing mixtures of dietary fibers (Bortoluzzi et al., 2017; Canfora et al., 2019). Some studies also showed that prebiotics, such as fructooligosaccharide and xylooligosaccharide, had an inhibitory effect on proteolysis (Lecerf et al., 2012). These results therefore suggested that inulin might decreasing the availability of amino acids for proteolytic bacteria, thus reducing ammonia production (Wang et al., 2019). Microbial metabolites derived from the fermentation of amino acids or proteins, mainly ammonia, amines, indole compounds, and hydrogen sulfide, are potentially detrimental to gut integrity, which could be responsible for SE-infection-related impairment of the mucosal barrier (Song et al., 2020). In the present study, we also found that fermentation of inulin increased butyrate and acetate production, which may decrease the energy sources from the fermentation of amino acids or protein for utilization by bacteria (Canfora et al., 2019). All these results indicated that the main strains responsible for proteolytic fermentation are *Bifidobacterium*, *Clostridium*, *Enterococcus*, *Lactobacillus*, *Pediococcus*, and *Streptococcus*, which may in turn be depleted by metabolizing the amino acids (Pugin et al., 2017), possibly helping to explain the decreases in abundances of *Bifidobacterium, Clostridium, Lactobacillus*, and *Streptococcus* in the SE group and the increases in inulin supplementation group in this study.

## Conclusion

The results of this study demonstrated that dietary supplementation of inulin reversed the decrease in the alpha diversity (Shannon index) and richness (Chao1 index) induced by SE-infection. Moreover, Inulin increased butyrate and acetate production, which may decrease the energy sources from the fermentation of amino acids or protein for utilization by microbe. Accordingly, the enrichments of metabolic pathways associated with amino acid metabolism and energy metabolism were reduced and the abundances of *Bifidobacterium, Clostridium, Lactobacillus*, and *Streptococcus* responsible for proteolytic fermentation were increased, which may be the main cause of inulin improving the cecal microbiome of SE-infected chickens.

## Data Availability Statement

The datasets presented in this study can be found in online repositories. The names of the repository/repositories and accession number(s) can be found below: NCBI PRJNA645511 and PRJNA645563.

## Ethics Statement

The animal study was reviewed and approved by the Animal Welfare Committee of the Institute of Animal Sciences (Chinese Academy of Agricultural Sciences, Beijing, China), Ethical approval regarding animal survival was given by the animal ethics committee of IAS-CAAS (Approval number: IASCAAS-AE20140615).

## Author Contributions

JS formulated the research question, performed the study, and drafted the manuscript. QL and RL contributed to suggestion of data analysis and interpretation. MZ and GZ contributed to the schedule the study. NE and JW contributed to study design and modify the manuscript. All authors contributed to the article and approved the submitted version.

## Conflict of Interest

The authors declare that the research was conducted in the absence of any commercial or financial relationships that could be construed as a potential conflict of interest.

## Footnotes

1 http://drive5.com/uparse/

2 http://rdp.cme.msu.edu/

3 www.illumina.com

4 http://bio-bwa.sourceforge.net/

5 https://github.com/jstjohn/SeqPrep

6 https://github.com/najoshi/sickle

7 https://sourceforge.net/projects/soapdenovo2/

8 http://metagene.cb.k.u-tokyo.ac.jp/

9 http://www.ncbi.nlm.nih.gov/Taxonomy/taxonomyhome.html/index.cgi?chapter=tgencodes#SG1

10 http://www.bioinformatics.org/cd-hit/

11 http://blast.ncbi.nlm.nih.gov/Blast.cgi

12 https://www.kegg.jp/

## Supplementary Material

Supplementary Figure 1 Venn diagrams of cecal microbiota at the operational taxonomic unit level.

Supplementary Figure 2 Hierarchical clustering tree of cecal microbiota at the operational taxonomic unit level.

Supplementary Figure 3 The microbiota differentially abundant at genus level among four groups in the cecum by One-way ANOVA analysis.

Supplementary Figure 4 Cecal microbiota differentially abundant at genus level between 0.5%InSE and 1%InSE groups by *t*-test.
