# Supplementary material for: Dietary Inulin Supplementation Modulates Short-Chain Fatty Acid Levels and Cecum Microbiota Composition and Function in Chickens Infected With Salmonella
Source: Front Microbiol. 2020 Dec 9;11:584380. doi: 10.3389/fmicb.2020.584380 (PMC7793945; doi:10.3389/fmicb.2020.584380)
Supplement: Supplementary Figure 1 — Venn diagrams of cecal microbiota at the operational taxonomic unit level. [file Data_Sheet_1.zip › Supplementary Material/Additional file/Table S1.docx]

**Table S1.** Composition and nutrient levels of basal diets (air-dried basis) (g/kg)

| **Item** | **Treatment** | | |
| --- | --- | --- | --- |
|  | **CON^1^** | **0.5% Inulin** | **1% Inulin** |
| Ingredients |  |  |  |
| Corn | 680.1 | 669.8 | 660.2 |
| Choline chloride | 0.3 | 0.3 | 0.3 |
| Soybean meal | 275.6 | 277.8 | 279.6 |
| Corn oil | 0 | 3 | 5.8 |
| Salt | 2 | 2 | 2 |
| Limestone powder | 4.8 | 4.8 | 4.8 |
| Calcium dihydrogen phosphate | 16 | 16 | 16 |
| Cystine | 3 | 3.1 | 3.1 |
| Methionine | 0.2 | 0.2 | 0.2 |
| Vitamin premix^2^ | 10 | 10 | 10 |
| Microelement premix^3^ | 5 | 5 | 5 |
| Feed grade silicondioxide/titanium | 3 | 3 | 3 |
| Inulin | 0 | 5 | 10 |
| Calculated nutrient level |  |  |  |
| ME, MJ/kg | 11.98 | 11.98 | 11.98 |
| CP | 181.0 | 181.0 | 181.0 |
| Available phosphorus | 4.0 | 4.0 | 4.0 |
| Calcium | 9.0 | 9.0 | 9.0 |
| Lysine | 9.1 | 9.0 | 9.1 |
| Methionine | 3.1 | 3.1 | 3.1 |
| Cystine | 6.2 | 6.2 | 6.2 |

^1^Control group without inulin supplementation.

^2^Vitamin premix provided the following per kilogram of diet: vitamin A, 12,000 IU; vitamin D3, 3500 IU; vitamin E, 25 IU; nicotinic acid, thiamin 60 mg; vitamin B12, 0.014 mg; calcium pantothenate, 20 mg; vitamin K3, 2.0 mg; thiamin, 2.0 mg; riboflavin, 8.0 mg; vitamin B6, 7.0 mg; folic acid, 0.8 mg; biotin, 0.2 mg.

^3^Microelement premix provided the following per kilogram of diet: Fe, 100 mg; Cu, 8 mg; Mn, 120 mg; Zn, 100 mg; I, 0.7 mg; Se, 0.3 mg.
