# Supplementary figures and images for: Dietary Inulin Supplementation Modulates Short-Chain Fatty Acid Levels and Cecum Microbiota Composition and Function in Chickens Infected With Salmonella
Source: Front Microbiol. 2020 Dec 9;11:584380. doi: 10.3389/fmicb.2020.584380 (PMC7793945; doi:10.3389/fmicb.2020.584380)

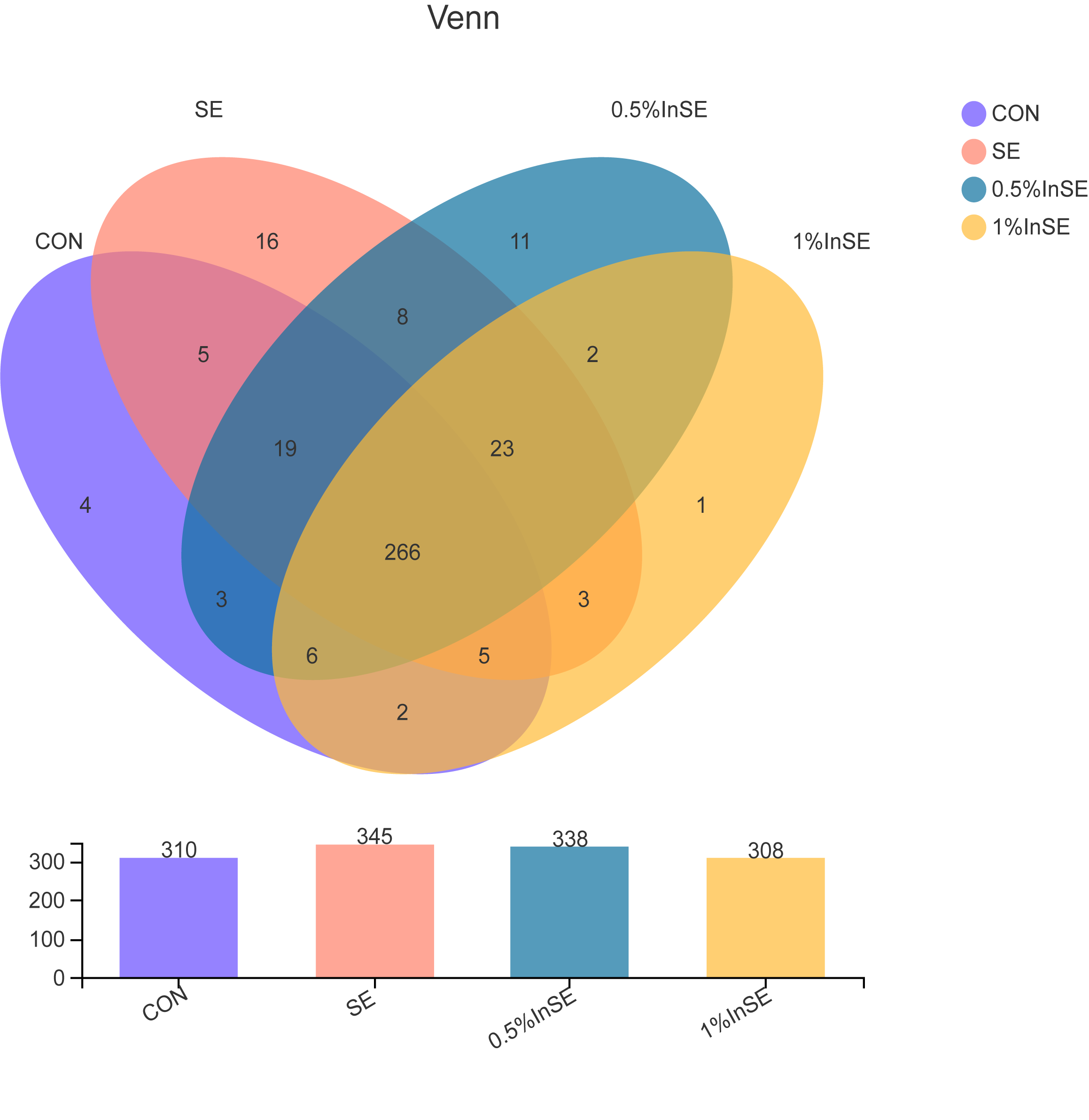

Supplement: Supplementary Figure 1 — Venn diagrams of cecal microbiota at the operational taxonomic unit level. [file Data_Sheet_1.zip › Supplementary Material/Additional file/Figure S1.tif]

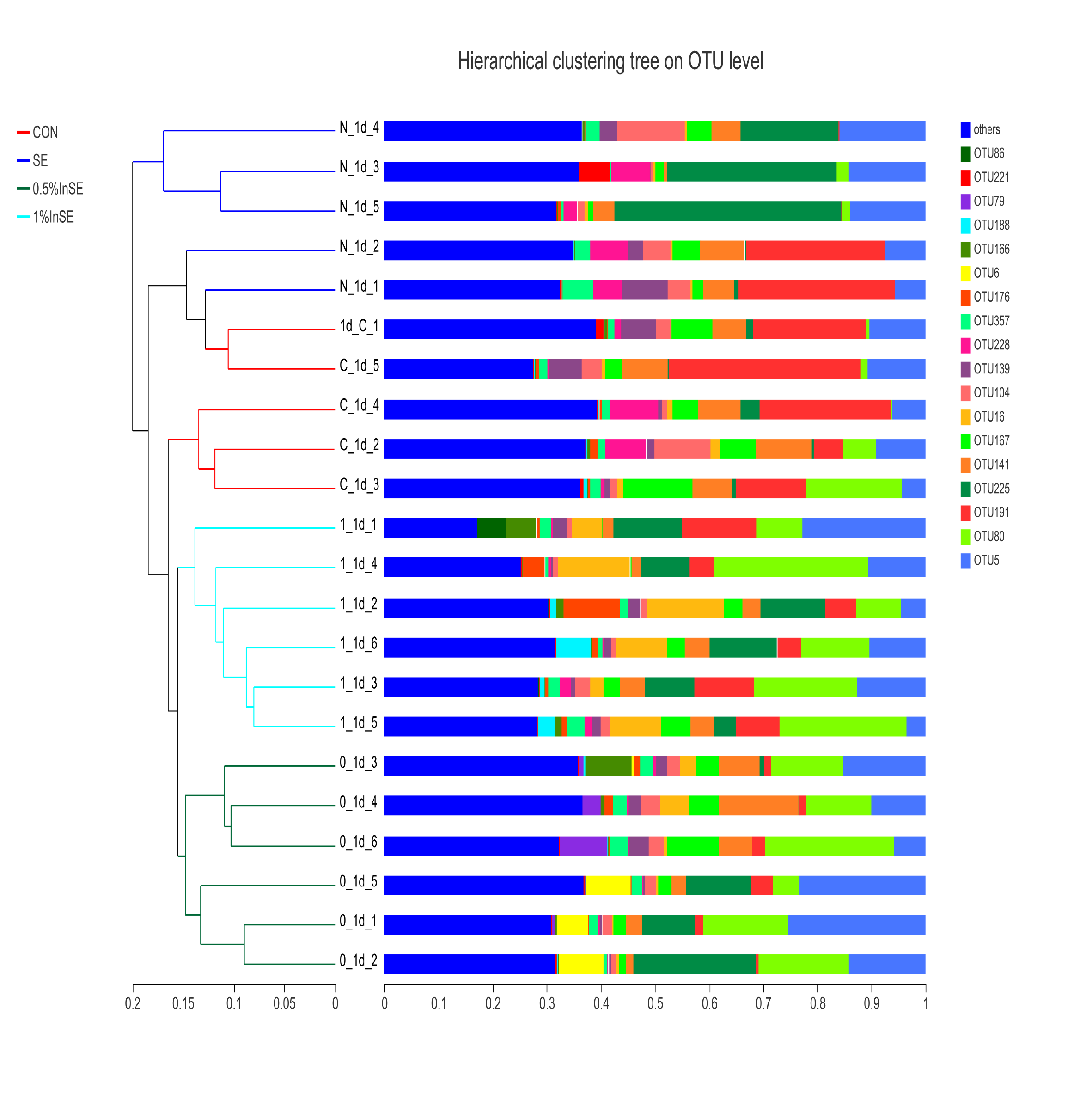

Supplement: Supplementary Figure 1 — Venn diagrams of cecal microbiota at the operational taxonomic unit level. [file Data_Sheet_1.zip › Supplementary Material/Additional file/Figure S2.tif]

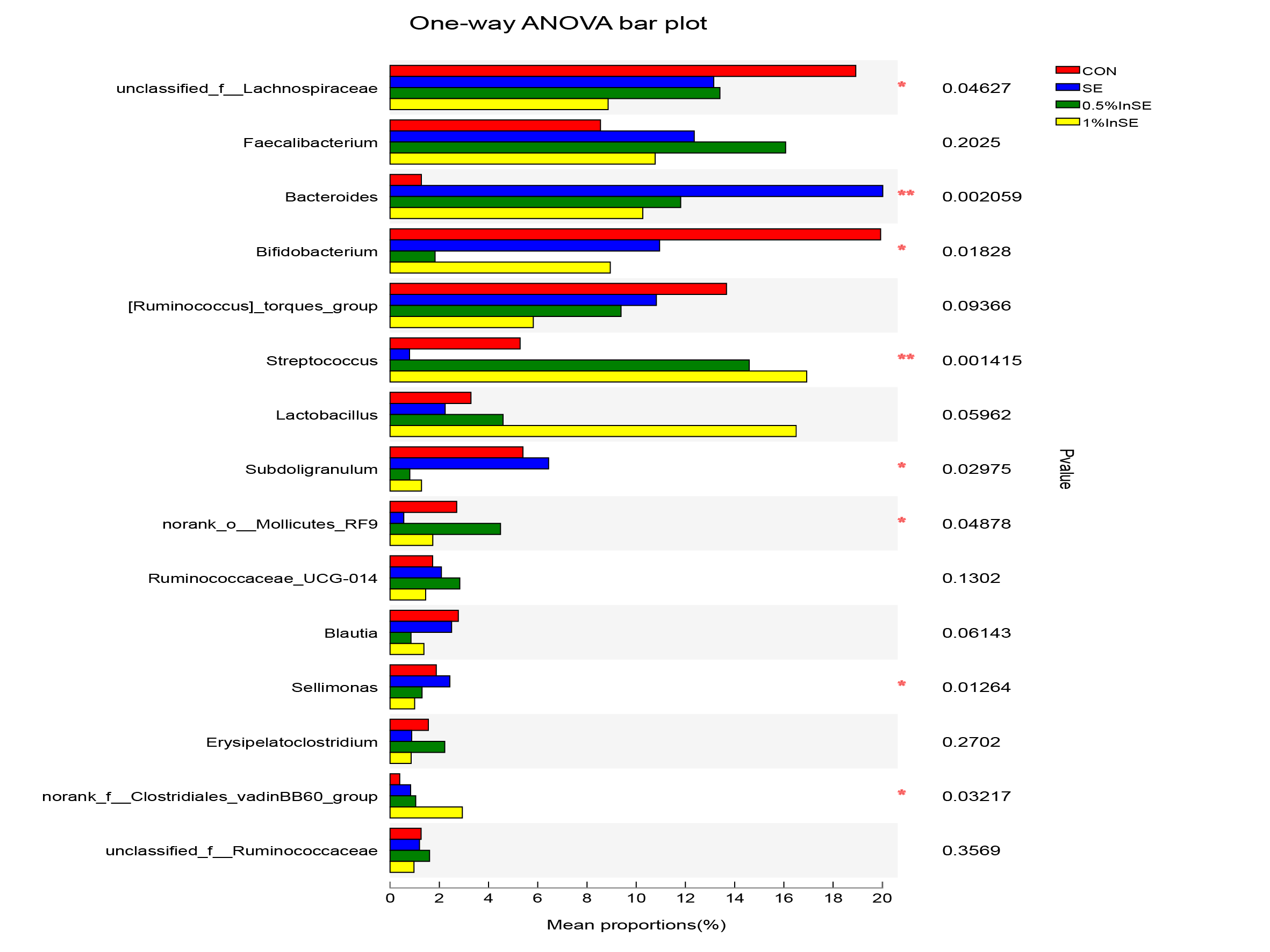

Supplement: Supplementary Figure 1 — Venn diagrams of cecal microbiota at the operational taxonomic unit level. [file Data_Sheet_1.zip › Supplementary Material/Additional file/Figure S3.tif]

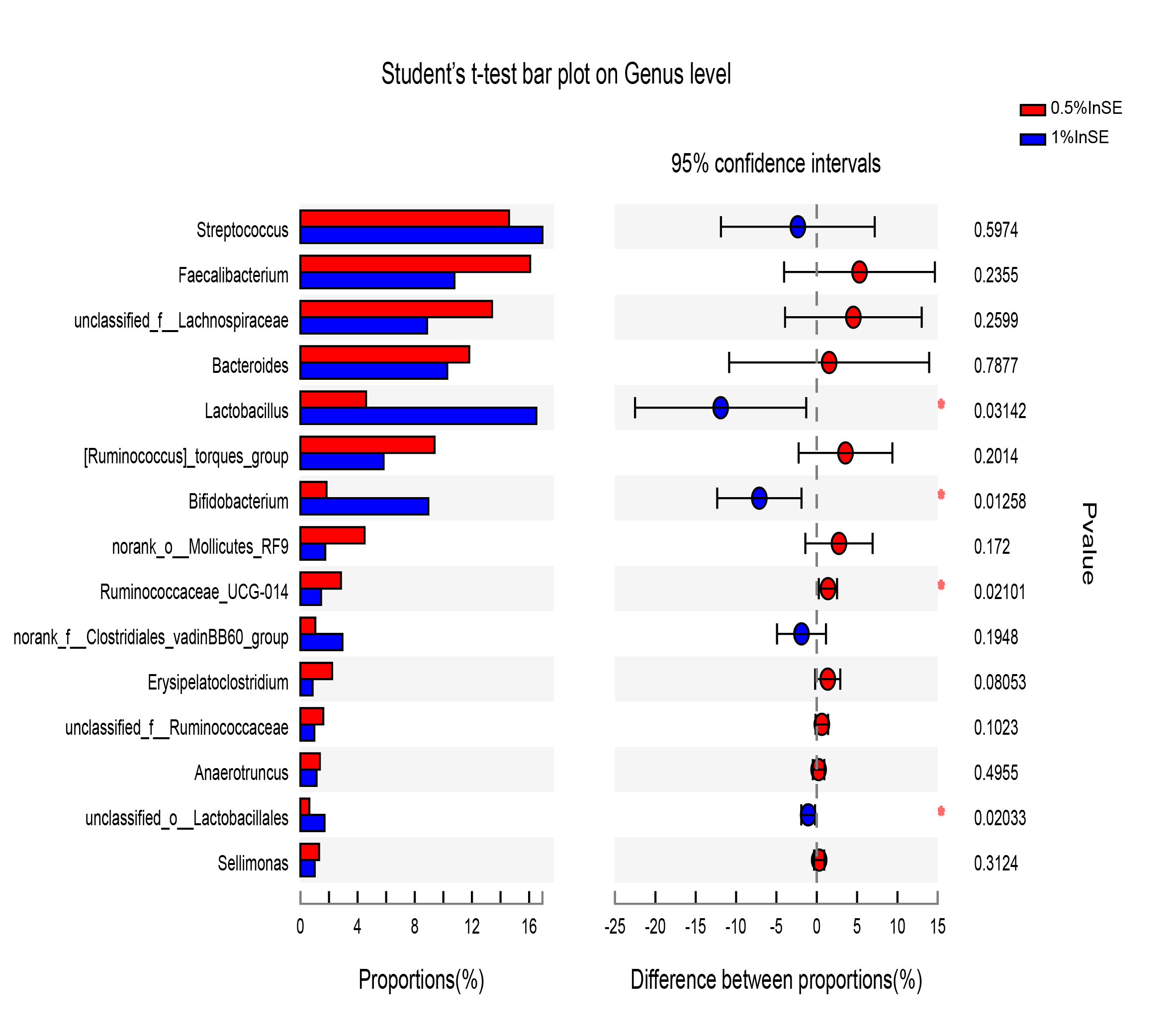

Supplement: Supplementary Figure 1 — Venn diagrams of cecal microbiota at the operational taxonomic unit level. [file Data_Sheet_1.zip › Supplementary Material/Additional file/Figure S4.tif]
